# The progression of doxorubicin-induced intestinal mucositis in rats

**DOI:** 10.1007/s00210-022-02311-6

**Published:** 2022-10-22

**Authors:** F. Kullenberg, K. Peters, C. Luna-Marco, A. Salomonsson, M. Kopsida, O. Degerstedt, M. Sjöblom, P. M. Hellström, F. Heindryckx, D. Dahlgren, H. Lennernäs

**Affiliations:** 1 Department of Pharmaceutical Biosciences, Uppsala University, 752 37 Uppsala, Sweden; 2 Department of Medical Cell Biology, Uppsala University, 751 23 Uppsala, Sweden; 3 Department of Medical Sciences, Uppsala University, 751 85 Uppsala, Sweden

**Keywords:** Chemotherapy-induced mucositis, Doxorubicin, Jejunum, Ki67, TUNEL, Permeability

## Abstract

Chemotherapy-induced intestinal mucositis is a severe side effect contributing to reduced quality of life and premature death in cancer patients. Despite a high incidence, a thorough mechanistic understanding of its pathophysiology and effective supportive therapies are lacking. The main objective of this rat study was to determine how 10 mg/kg doxorubicin, a common chemotherapeutic, affected jejunal function and morphology over time (6, 24, 72, or 168 h). The secondary objective was to determine if the type of dosing administration (intraperitoneal or intravenous) affected the severity of mucositis or plasma exposure of the doxorubicin. Morphology, proliferation and apoptosis, and jejunal permeability of mannitol were examined using histology, immunohistochemistry, and single-pass intestinal perfusion, respectively. Villus height was reduced by 40% after 72 h, preceded at 24 h by a 75% decrease in proliferation and a sixfold increase in apoptosis. Villus height recovered completely after 168 h. Mucosal permeability of mannitol decreased after 6, 24, and 168 h. There were no differences in intestinal injury or plasma exposure after intraperitoneal or intravenous doxorubicin dosing. This study provides an insight into the progression of chemotherapy-induced intestinal mucositis and associated cellular mucosal processes. Knowledge from this *in vivo* rat model can facilitate development of preventive and supportive therapies for cancer patients.

**Supplementary Information:**

The online version contains supplementary material available at 10.1007/s00210-022-02311-6.

## Introduction

Global cancer incidence reached 19 million in 2020 (Sung et al. 2021). This number is expected to increase as mortality rates from stroke and cardiovascular disease decrease in an aging population (Bray et al. 2021). Cancer is typically treated with chemotherapy, which inhibits cell growth and division. This also severely affects rapidly proliferating non-tumor tissue, such as myeloid and lymphoid tissues, gonads, and intestinal mucosa (van der Zanden et al. 2020). Damage to the latter gives rise to chemotherapy-induced intestinal mucositis (CIM), which is a complex gastrointestinal toxicity affecting approximately 40% of patients treated with chemotherapeutics (Pico et al. 1998; Sonis et al. 2015; McCullough 2017; Sougiannis et al. 2021). High-dose myeloablative chemotherapies such as busulfan, etoposide, and doxorubicin (DOX) have a CIM incidence rate over 90% (McCullough 2017; Eduardo et al. 2019). Occurrence of severe gastrointestinal complications depends on several factors, including the immunological status of the patient, the type of drug, and the dosing schedule.

Doxorubicin is an anthracycline-type of chemotherapeutic commonly used for treating lymphoma, and breast, gastric, ovarian, sarcoma, and primary liver cancers. It induces apoptosis and other types of programed cell death, by (a) intercalating into nucleus DNA (thereby inhibiting biosynthesis of macromolecules and disrupting topoisomerase II-mediated DNA repair), and (b) intracellular generation of reactive oxygen species (Gewirtz 1999; Marinello et al. 2018; Kullenberg et al. 2021). In addition, anthracyclines are associated with mitochondrial dysfunction, which contributes to the anti-cancer effect, but also affects off-target organs such as the heart, bone-marrow, and gastrointestinal tract (Gewirtz 1999; Jones et al. 2021).

Adverse effects associated with CIM, such as diarrhea, ulceration, pain, nausea, sepsis, and organ dysfunction, reduce the quality of life and are potentially fatal for susceptible cancer patients. This often necessitates dose-reduction or discontinuation of cancer treatment, and increases healthcare costs (Dahlgren et al. 2021b; Rodrigues-Oliveira et al. 2021). Despite progress in many cancer treatments, CIM remains a significant, common clinical challenge with a need for safe and effective supportive treatments. A prerequisite for better treatments is an improved understanding of the underlying mechanisms of CIM as well as its progression over time in preclinical models. These results can translate to improved patient care in the future (Sonis 2009).

Chemotherapy-induced mucositis is initiated by damage to proliferative cells in the crypts of the epithelium. This is followed by a primary damage response characterized by activation of pro-apoptotic and pro-inflammatory signaling pathways that jointly contribute to intestinal stem cell death (Bowen et al. 2019). These signaling pathways are then amplified, which may lead to ulceration and tissue inflammation. Morphologically, CIM is characterized by a reduction in villus height, and functionally, by dysregulation of the mucosal barrier and its secretory and absorptive homeostasis (Tonneau et al. 2021). This is followed by an increase in epithelial proliferation, differentiation, and maturation, while the activated inflammatory pathways return to baseline (Sonis et al. 2015). Recently, we reported experimental results based on this in vivo rat model where villus atrophy was determined to be similar to that observed in this study following single-dose administration of five different chemotherapeutics (doxorubicin, idarubicin, methotrexate, 5-fluorouracil, and irinotecan) at clinically relevant doses and combinations. However, diarrhea was only observed for irinotecan and idarubicin and it was concluded that small intestinal villus atrophy itself was not predictive of diarrhea. These local intestinal processes will aid to investigate other mechanisms along the intestine, as an improved understanding of this relationship is expected to be crucial for the development of supportive treatments for CIM and diarrhea induced by cancer drugs (Dahlgren et al. 2022).

While several previous studies show substantial villus atrophy 72 h after chemotherapy in rodents (Kissow et al. 2012; Sukhotnik et al. 2014), the progression of CIM and the link to mucosal functions such as intestinal permeability are generally absent from these studies (Sun et al. 1998; Dekaney et al. 2009; Kaczmarek et al. 2012). Furthermore, the balance between mucosal proliferation and cell death and the relationship to functional and morphological changes need to be better understood for successful development of supportive treatments (Sonis et al. 2015; Eduardo et al. 2019). Another issue is that in most rodent CIM studies, chemotherapy is delivered intraperitoneally (IP) (Sun et al. 1998; Dekaney et al. 2009; Kaczmarek et al. 2012; Kissow et al. 2012; Sukhotnik et al. 2014). This can be problematic when studying intestinal damage caused by chemotherapeutics, which are typically delivered intravenously (IV). This is especially so for DOX, a drug known to cause necrosis upon contact with soft tissue (Reilly et al. 1977; Boschi and Rostagno 2012).

The main objective of this rat in vivo study was to determine the effect of DOX on the initiation and progress of CIM by monitoring morphological, cellular, and functional, jejunal changes over 7 days. The secondary objective was to compare the severity of CIM to the plasma exposure of DOX, following IP and IV single-dose administration.

## Materials and methods

### Chemicals and solutions

Accustain formalin solution (10%, neutral buffered), ethanol, thiobutabarbital sodium (Inactin), dimethyl sulfoxide, and phosphate buffered saline tablets (PBS, pH 7.4) were purchased from Sigma-Aldrich (Darmstadt, Germany). Sodium phosphate dibasic dihydrate (Na_2_HPO_4_∙2H_2_O), potassium dihydrogen phosphate (KH_2_PO_4_), sodium hydroxide, methanol, and sodium chloride were purchased from Merck KGaA (Darmstadt, Germany). Invitrogen RNAlater Stabilization Solution was purchased from Fisher Scientific (Pittsburgh, PA, USA). All solvents were HPLC-grade or higher and water was of ultra-pure grade (Milli-Q). Doxorubicin (DOX) hydrochloride was purchased from Toronto Research Chemicals, Canada. Transferrin Ki67 antibody (ab16667), horseradish peroxidase–DAB (3,3′-diaminobenzidine) Detection IHC Kit (ab64261) and terminal deoxynucleotidyl transferase dUTP nick-end labeling (TUNEL) Assay Kit–HRP-DAB (ab206386) were purchased from Abcam, Cambridge, UK. Doxorubicinol (DOXol) trifluoroacetate, as well as the internal standards (IS) ^13^C, ^2^H_3_-DOX trifluoroacetate (DOX IS) and ^13^C, ^2^H_3_-DOXol trifluoroacetate (DOXol IS), was purchased from Alsachim (Illkirch-Graffenstaden, France). ^3^H-labeled mannitol was purchased from PerkinElmer Life Sciences (Boston, MA, USA). An isotonic (290 mOsm) phosphate-buffered (pH 6.5, 8 mM) solution was prepared for the perfusion experiments. Osmolality was determined by freezing-point decrement using a Micro Osmometer (Model 3MO; Advanced Instruments, Needham Heights, MA, USA). Thiobutabarbital sodium was prepared at 500 mg/mL in deionized water. Stock solutions (100 mg/mL) were prepared by dissolving DOX hydrochloride in dimethyl sulfoxide. The stock solution was then diluted to 5 mg/mL in physiological saline, which was the administered concentration. ^3^H-mannitol was prepared at 2.5 µCi/mL and 0.5 µCi/mL in saline for bolus dosing and continuous infusion, respectively.

### Animals

This animal study was approved by the local ethics committee for animal research (Dnr 5.8.18–06,777/2020) in Uppsala, Sweden. Male Wistar Han IGS rats (strain code 273) from Charles River Co. (Germany and France) with body weight 230–470 g (age 6–14 weeks) were used. All animals were allowed to acclimatize for at least 1 week in the Animal Department prior to the start of the experiment and allowed water and food ad libitum. Housing conditions were 21–22 °C at a 12–12 h light–dark cycle. Animals were evaluated for general welfare twice daily by the Animal Department staff. Exclusion/termination was based on weight loss (> 20%) and visual evaluation.

### Study design

There were eight groups in this study: one saline (with 5% DMSO) IV control, six DOX IV, and one DOX IP. At different time points after dosing, rats were single-pass perfused while anesthetized (thiobutabarbital sodium IP, 180 mg/kg) to determine jejunal mucosal permeability. Immediately after the end of the perfusion, jejunal tissue samples were taken for morphological, proliferative, apoptotic, immunological, and biochemical assays (see “Single-pass intestinal perfusion and collection of samples” section).

The control group (*n* = 6) was administered physiological saline containing 5% DMSO (as solvent control) and evaluated after 6 h. To study the time-dependent effects of DOX, an IV dose of 10 mg/kg was selected for clinical translation (Nair and Jacob 2016). The rats were divided into four groups (*n* = 6) that were examined at 6, 24, 72, or 168 h post dose.

In two groups, the impact of 2 days subsequent 10 mg/kg DOX IV doses on intestinal toxicity were investigated; one 72 h after the first injection (double dose 1, *n* = 3) and one after 96 h (double dose 2, *n* = 2). This part of the study was terminated early due to severe side-effects.

In a separate 72-h group (*n* = 6), an IP injection of DOX (10 mg/kg) was given to investigate if the administration route had any effect on intestinal toxicity and the plasma pharmacokinetics of DOX and its main metabolite DOXol. The rats were sedated (thiobutabarbital sodium IP, 180 mg/kg) and dosed with 10 mg/kg DOX via IV or IP (*n* = 6 per group). Arterial blood was sampled at 5, 10, 20, 40, 60, 120, 240, and 360 min after dosing, centrifuged (miniSpin, Eppendorf, Hamburg, Germany) at 5000 × *g* for 5 min, and the plasma collected and stored at − 20 °C until analysis.

### Single-pass intestinal perfusion and collection of samples

The effect of DOX on intestinal permeability after IV and IP dosing was examined with an intestinal single-pass perfusion model, as reported in detail elsewhere (Dahlgren et al. 2021a). After intestinal surgery, ^3^H-mannitol was administered IV as a bolus of 0.25 µCi (0.1 mL), followed by a continuous IV infusion at a rate of 0.5 µCi/h (1 mL/h) throughout the experiments of 120 min. For the first 45 min following surgery, the jejunal segment was single-pass perfused with phosphate-buffered perfusate solution (pH 6.5, 8 mM, 37 °C) to allow for cardiovascular, respiratory, and intestinal stabilization prior to permeability assessment. The length and wet tissue weight of excised jejunal segment in each rat was determined after the experiment.

Following a 45-min stabilization period, control buffer was perfused during 75 min. Perfusate leaving the intestinal segment was collected and weighed at 15-min intervals throughout the experiments. Blood samples (< 0.3 mL) were drawn from the femoral artery at the start (*t* = 0 min) and at the end (*t* = 75 min) of the perfusions. The blood samples were centrifuged (miniSpin) at 5000 × *g* for 5 min within 10 min, and the plasma and perfusates were analyzed for ^3^H activity. The luminal single-pass perfusion rate was at all times 0.2 mL/min throughout the study, controlled via a peristaltic pump (Gilson Minipuls 3, Le Bel, France). The body temperature of the animals was monitored throughout the perfusion experiments and kept stable at 37.5 ± 0.5 °C.

After completion of the intestinal perfusion, the rats were dissected and samples were taken from the jejunum, both for histology and real-time quantitative polymerase chain reaction (RT-qPCR) analyses. The samples for RT-qPCR were submerged in RNAlater (Fisher Scientific) at room temperature for approximately 30 min, and then stored at − 80 °C until analysis. The histology samples were fixed in 10% formalin for 24 h, after which they were moved to 70% ethanol and then embedded in paraffin for subsequent histological and immuno-histological analyses.

### Bioanalytical method for quantification of plasma concentrations of DOX and DOXol

The assay for DOX and DOXol quantification was adapted from an earlier report (Kullenberg et al. 2021). Briefly, an ACQUITY UPLC I-Class system coupled to a TQS micro tandem mass spectrometer (Waters Corporation, Milford, MA, USA) was used to quantify plasma concentrations of both DOX and its main metabolite DOXol. The chromatography was identical to the published method (Kullenberg et al. 2021). The ionization technique was positive electrospray and the positive capillary voltage was 1.5 kV. The desolvation temperature was 500 °C; the cone and desolvation gas flows were 100 L/h and 800 L/h, respectively. The quantification was performed in multiple reaction monitoring mode with the transitions m/z 544 → 397 for DOX (collision energy 13 eV, cone voltage 40 V), 546 → 399 for DOXol (collision energy 13 eV, cone voltage 30 V), 548 → 401 for DOX IS (collision energy 13 eV, cone voltage 40 V), and 550 → 403 for DOXol IS (collision energy 13 eV, cone voltage 30 V).

Stock solutions of analytes and isotopically labeled internal standards were prepared in methanol (1 mg/mL). From these, working standards containing DOX and DOXol were diluted in methanol and stored in amber vials at − 20 °C. All samples were prepared in 96-well collection plates (350 µL, Waters Corporation) and calibration curves were constructed in untreated (blank) rat plasma. For IP samples, 50 µL rat plasma was spiked with 10 µL of the working standard solutions. To precipitate plasma protein, chilled acetonitrile (190 µL) containing internal standards (50 nM) was added to the IP samples before storage overnight at − 20 °C. IV samples were prepared in a similar fashion but the volumes were 100 µL rat plasma, 25 µL working standard solution, and 375 µL acetonitrile containing internal standards (400 nM). The following day, samples were centrifuged at 644 × *g* for 3 min at 4 °C. Portions of the supernatants (50 µL for IP and 100 µL for IV samples) were transferred to a new 96-well plate and dried in a water bath (37 °C) under a gentle stream of nitrogen from a 3D-printed manifold. The residuals were dissolved in mobile phase A (50 µL for IP and 100 µL for IV) and then injected (5 µL for IP and 10 µL for IV samples) into the instrument. Linear calibration curves for DOX were constructed between 14 and 25,425 nM (*R*^2^ > 0.99) for IV samples and 12 to 4861 nM (*R*^2^ > 0.96) for IP samples. Linear calibration curves for DOXol were constructed between 16 and 25,574 nM (*R*^2^ > 0.99) for IV samples and 14 to 4567 nM (*R*^2^ > 0.96) for IP samples. Consequently, the LLOQ was 12 nM for DOX and 14 nM for DOXol.

IP and IV rat plasma samples of unknown concentration were treated exactly as the calibration samples, except that the added methanol did not contain any spiked DOX or DOXol. The selectivity was demonstrated using matrix blanks injected randomly throughout the sample runs. The data were processed using a linear curve fit (weighting factor of 1/ ×) of the peak area ratio (analyte:internal standard) as a function of the analyte concentration. All the collected data were processed using TargetLynx as part of MassLynx V4.1 (Waters Corporation). Quality control (QC) samples of DOX and DOXol at 250, 5000, and 25,000 nM were within 15% of the nominal value.

### Immunohistochemical and morphological analysis of excised jejunal samples

**Fig. 1:**
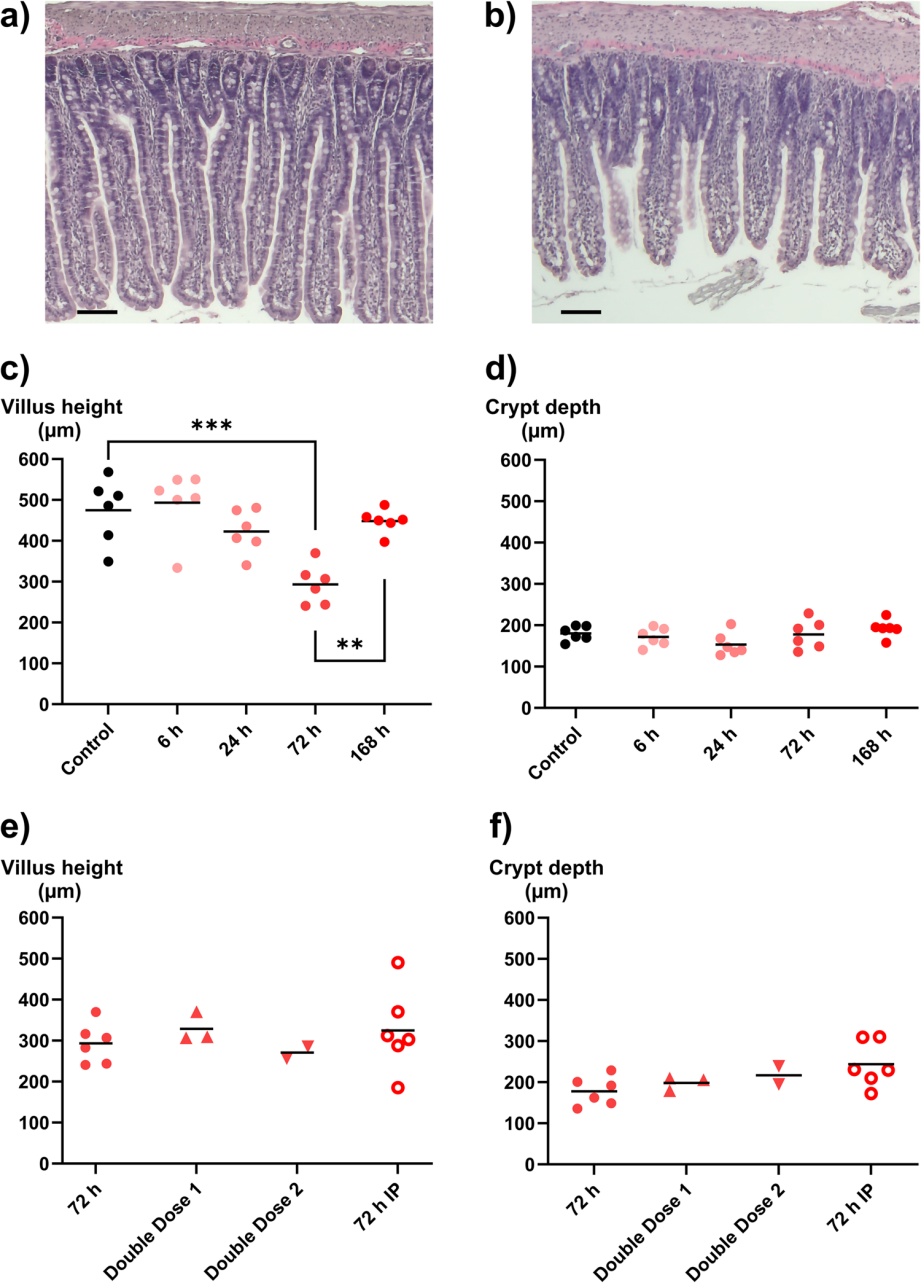
**a**–**f** Effect of time, administration route, and single or double doses of doxorubicin (DOX, 10 mg/kg) on villi height and crypt depth. Two example images are shown: **a** from a control animal and **b** at 72 h after DOX injection. Villus height is in (**c**) and (**e**), and crypt depth is in (**d**) and (**f**). The effect of time after a single IV dose of DOX is seen in (**c**) and (**d**), while **e** and **f** show effects of different administration routes (IV and IP) and number of DOX doses (one or two). All DOX was given via IV unless otherwise specified. Each symbol (circles, squares, and triangles) represents the average value from a single animal based on ten separate determinations of its villi or crypts, and the black line signifies the group average. The statistical data analysis was performed with an ANOVA analysis with Šidák’s multiple comparisons post hoc test, and comparisons with *p* < 0.05 were considered significant, indicated by a star (*p* < 0.05), two stars (*p* < 0.01), or three stars (*p* < 0.005). Scale bars are 100 µm

Formalin-fixed, paraffin-embedded tissue blocks were sectioned using a microtome at a thickness of 5 µm. Jejunal tissue slides were then deparaffinized and stained with hematoxylin and eosin, according to standard practice, then dehydrated and mounted. Images were acquired using a Zeiss Axio Vert microscope equipped with a Zeiss Axiocam 208 color camera and Zeiss A-Plan 5 × /0,25 Ph1 objective. The Zeiss Zen Blue 4.3 software was used to save and transfer the pictures. To evaluate the overall morphology and intestinal damage, villus height and crypt depth were measured on hematoxylin–eosin-stained slides, using Fiji ImageJ (Fig. 1).

To detect cell proliferation, immunohistochemistry was performed using antibody and the horseradish peroxidase–DAB Detection IHC kit, following manufacturer’s guidelines (supplementary information A). In short, slides were deparaffinized, washed in PBS with Tween-20 and endogenous peroxidase activity was blocked using hydrogen peroxide. A DIVA-decloaking chamber was used to retrieve crosslinked antigens and non-specific background staining was blocked using the kit’s protein block solution. Primary Ki67 antibody was added in a 1:1000 dilution of PBS-Tween and incubated for 1 h at 37 °C. Slides were subsequently incubated for 10 min with biotinylated goat anti-rabbit antibodies and streptavidin peroxidase at room temperature. Finally, DAB was added and applied to the jejunum tissue for 2 min and rinsed. Washing in between steps was done with PBS. Finally, slides were counterstained with hematoxilin, dehydrated, and mounted.

To detect apoptosis, the samples were stained with a TUNEL assay kit according to the manufacturer’s instructions. Cells stained with the TUNEL assay are not specifically undergoing apoptosis, but rather programmed cell death in general (Gavrieli et al. 1992; Mirzayans and Murray 2020). However, in this study, we refer to the stained cells as apoptotic in concurrence with previous studies (Keefe et al. 2000; Gibson et al. 2003; Al‐Dasooqi et al. 2011). Images were acquired using a Zeiss Axio Vert microscope equipped with a Zeiss Axiocam 208 color camera and Zeiss A-Plan 10 × /0,25 Ph1 objective. The Zeiss Zen Blue 4.3 software was used to save and transfer the pictures. The images were processed using in an ImageJ macro to automatically quantify the amount of DAB staining (supplementary information B and C). For TUNEL staining, the images were processed using ImageJ to quantify the number of stained cells per crypt by manually counting the number of stained cells in the crypt region per captured image and dividing by the number of crypts detected in the same image.

### mRNA-expression via real-time quantitative polymerase chain reaction (RT-qPCR)

Five milligrams of intestinal tissue from each rat was homogenized in 350 µL TRK Lysis buffer (15131GF24, Omega Bio-tek, Inc., Norcross, Georgia, USA), using syringes. The E.Z.N.A. Total RNA Kit II isolation kit (R6834-02, Omega Bio-tek, Inc., Norcross, Georgia, USA) was subsequently used to isolate RNA, following the manufacturer’s guidelines. All centrifugations were performed at 10,000 × g. Quality and quantity of RNA was evaluated using nanodrop, by measuring the absorbance ratio at both 260/280 and 230/260 nm and reading the full absorption spectrum. To generate complementary DNA, the 50RXN SuperScript IV VILOMaster Mix (10,459,604, Thermo Fisher Scientific, Waltham, MA, USA) was used and applied according to the manufacturer’s protocol. Four microliters of master mix from the kit, 1 µL nuclease-free water, and 15 µL of diluted RNA samples were added to each well and incubated at 46 °C for 20 min in a Thermal Cycler Range (Techne Prime, Bibby Scientific, Staffordshire, UK). Primers were designed using ncbi-Primer Blast and ordered from Thermo Fisher Scientific (Table S2). A Fast SYBR Green Master Mix (Ref: 4,385,612, Applied Biosystems by 10,459,604, Thermo Fisher Scientific) was used according to the manufacturer’s guidelines. Quantification of gene expression was done using QuantStudio 5 (Applied Biosystems by Thermo Fisher Scientific). Normalization of mRNA-expression was performed with *GAPDH* and *beta-actin* as reference genes*.* The average CT-value of the two or three technical duplicates for each sample was calculated to determine fold change using the delta-delta-CT approach.

### Determination of blood-to-lumen jejunal mucosal ^3^H-mannitol clearance

Luminal perfusion solutions leaving the segment and blood plasma samples (at *t* = 0 and *t* = 75) were mixed with an appropriate scintillation cocktail (Pico-Fluor Plus, Perkin Elmer Life Sciences, Boston, MA, USA) and analyzed for ^3^H activity (cpm) in a liquid scintillation analyzer (Tri-Carb 2910 TR, Perkin Elmer Life Sciences, Boston, MA, USA). A linear regression analysis of the plasma samples was made to calculate a corresponding plasma value for each time point at which a perfusate sample was taken. The blood-to-lumen clearance (CL) of ^3^H-mannitol was calculated using Eq. (1):

1 $$CL=\frac{{C}_{perfusate}*{Q}_{in}}{{C}_{plasma}*{m}_{tissue}}*100$$

where C_perfusate_ and C_plasma_ are the activities (cpm/mL) in the perfusate and plasma, respectively, Q_in_ is the flow rate (mL/min) into the segment, and m_tissue_ is the weight of the perfused tissue. CL is expressed as mL/min/100 g wet tissue weight (Nylander et al. 1989). The blood-to-lumen clearance in Eq. 1 represents the small intestinal barrier function (permeability) (Nylander et al. 1991; Krugliak et al. 1994). The average CL for each rat over all time points was then calculated.

### Non-compartmental analysis of pharmacokinetic data

The PK parameters for DOX were calculated from the plasma concentration–time profile of DOX by a non-compartmental analysis (NCA) method using the ncappc 0.30 package in R 4.1.0; see supplementary information D and supplementary Table S1. The area under the plasma concentration–time curve (AUC_0–6 h_) to the last measured concentration was calculated by using the linear trapezoidal rule for the positive or zero local slopes (increasing concentration or at the peak), while the log-linear approximation method was used to estimate of the area under a curve at the negative local slope (decreasing concentration). The half-life (t_1/2_) was estimated using the terminal rate constant obtained by log-linear regression analysis of the last three concentration–time points. Volume of distribution (V_d_) was estimated based on AUC.

### Statistical analysis

The statistical data analysis was performed with an ANOVA analysis with Šidák’s multiple comparisons post hoc test, and comparisons with *p* < 0.05 were considered significant. The difference in t_1/2_, V_d_, and AUC_0–6 h_ from the NCA of the two administration routes were compared using a student’s *t*-test. All comparisons were tested for normality of residuals and equality of group variance with the tests Shapiro–Wilk and Brown–Forsythe, respectively. If the group variance was not equal, the regular ANOVA analysis was replaced with a Brown–Forsythe ANOVA test with a Dunnet T3 post hoc test. If a non-normal distribution was indicated, the non-parametric Kruskal–Wallis test with Dunn’s multiple comparisons post hoc test was used.

All statistical tests and graphs were performed in GraphPad Prism 9.0.0 (GraphPad Software, San Diego, CA, USA).

## Results

### Mucosal morphology

The effects of DOX on mean villus height and crypt depth in the jejunum, as well as example images, are shown in Fig. 1a–f. The villus height following a single IV dose of DOX (10 mg/kg) was reduced from 475 ± 80 μm to 293 ± 49 μm (*p* < 0.005) at 72 h. The villus height recovered to 448 ± 29 µm at 168 h after DOX IV dosing (Fig. 1c). There was no clear effect on crypt depth for any time point in any dosing group (Fig. 1d).

No differences in villus height or crypt depth were found between DOX IV and IP dosing at 72 h, nor between the single or double IV doses (Fig. 1e–f). Due to high mortality during anesthesia in the two dosing schedules with consecutive dosing, these were aborted before these groups were completed (*n* = 3 and 2, double doses 1 and 2, respectively).

### Proliferation and programmed cell death (apoptosis)

**Fig. 2:**
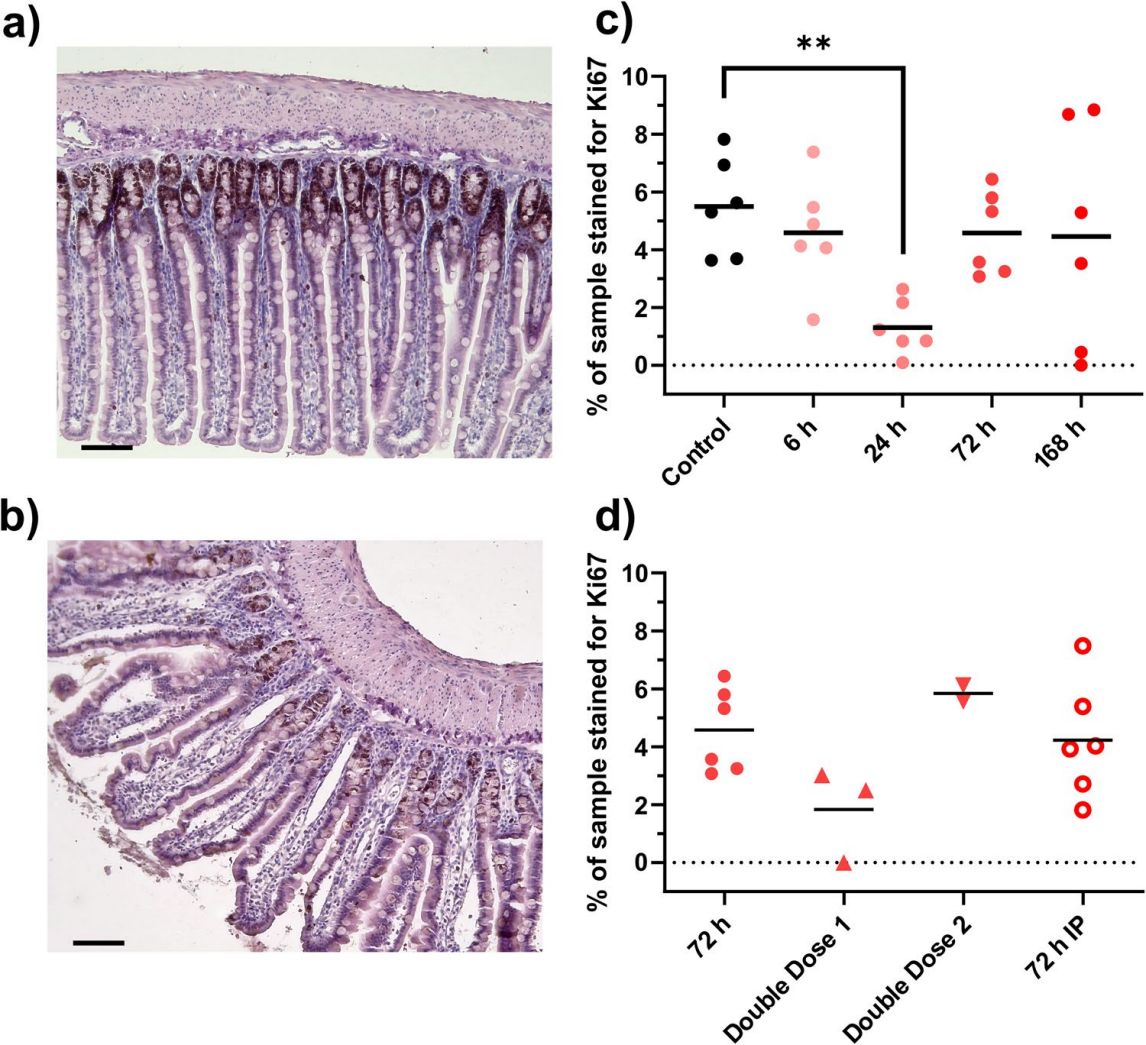
**a**–**d** Proliferation, detected with Ki67 staining, in jejunum samples after dosing of doxorubicin (DOX, 10 mg/kg). Two examples of tissue images are shown: **a** control animal, and **b** 24 h after DOX injection. The effect of time after a single IV dose of DOX is seen in (**c**), while **d** shows effects of different administration routes (IV and IP) and number of DOX doses (one or two). All DOX was given via IV unless otherwise specified. Each symbol (circles, squares, and triangles) represents the average value from a single animal, and the black line signifies the group average. The statistical data analysis was performed with an ANOVA analysis with Šidák’s multiple comparisons post hoc test, and comparisons with *p* < 0.05 were considered significant, indicated by a star (*p* < 0.05) or two stars (*p* < 0.01). Scale bars are 100 µm

**Fig. 3:**
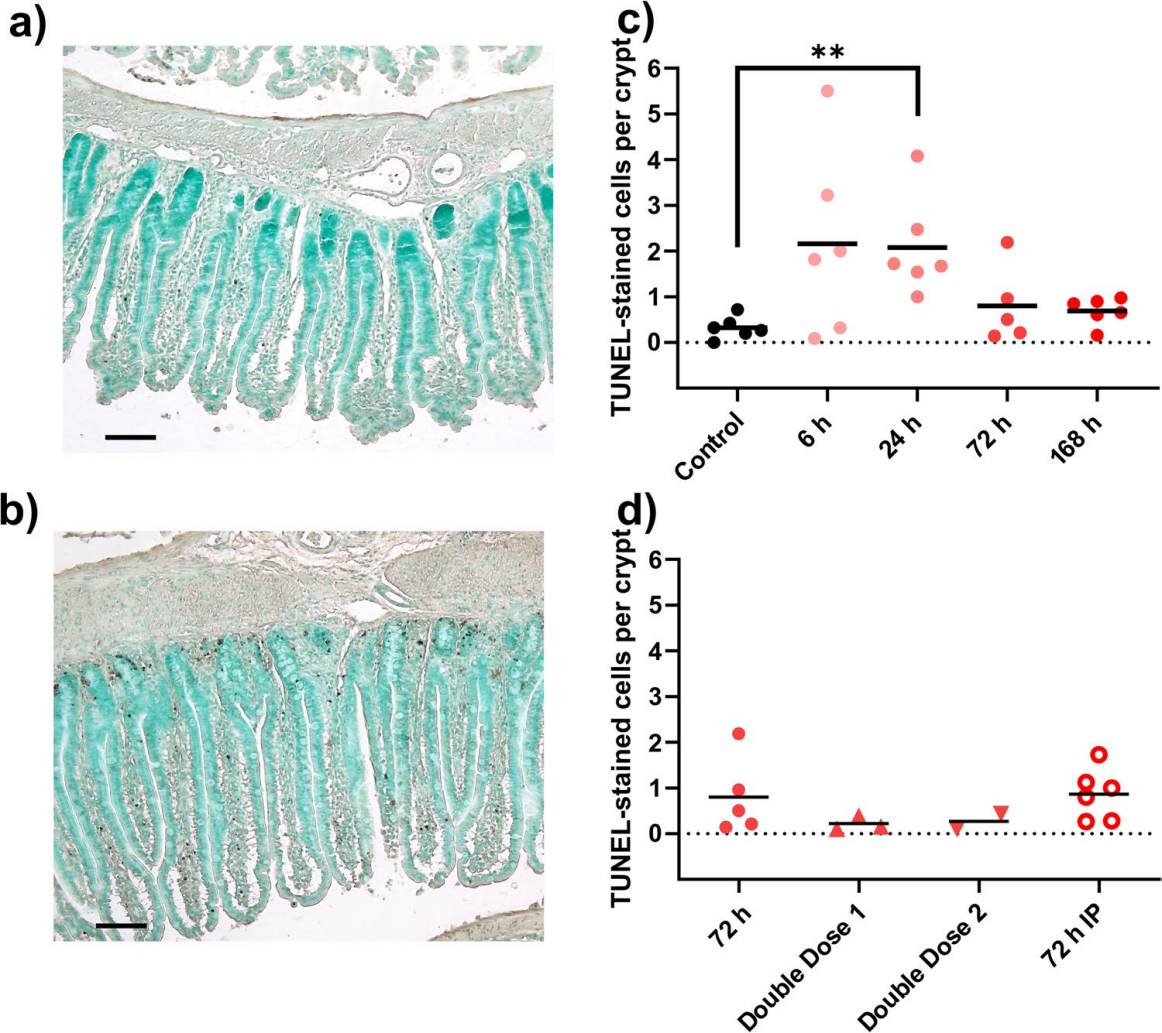
**a**–**d** Number of TUNEL stained cells in jejunum samples, representing apoptosis, after dosing of doxorubicin (DOX, 10 mg/kg). Two example images, are shown: **a** control animal, and **b** 24 h after DOX injection. The effect of apoptosis over time after a single IV dose of DOX is seen in (**c**), while **d** shows effects of different administration routes (IV and IP) and number of DOX doses (one or two). All DOX was given via IV unless otherwise specified. Each symbol (circles, squares, and triangles) represents the average value from a single animal, and the black line signifies the group average. The statistical data analysis was performed with a Kruskal–Wallis test with Dunn’s multiple comparisons post hoc test, and comparisons with *p* < 0.05 were considered significant, indicated by a star (*p* < 0.05) or two stars (*p* < 0.01). Scale bars are 100 µm

Staining with Ki67 antibodies revealed that the degree of proliferation in the crypts decreased. As seen in the example images, the control (Fig. 2a) has more DAB staining than the 24 h sample (Fig. 2b). This was also seen in the quantification, where percent staining was decreased by 75% (*p* < 0.005) 24 h after DOX IV dosing, and then recovered compared to baseline at 72 and 168 h (Fig. 2c). There was an opposite trend for apoptosis with the TUNEL staining, where the control (Fig. 3a) had less staining than the 24-h sample (Fig. 3b). This is also seen in the quantification, where the number of stained cells per crypt was six times higher (*p* < 0.05) at 24 h after DOX dosing, and again recovered compared to baseline at 72 and 168 h (Fig. 3c). No significant differences in proliferation or apoptosis were between IV and IP dosing, nor between the single or double IV doses (Figs. 2d and 3d).

### Plasma concentration–time profile

**Fig. 4:**
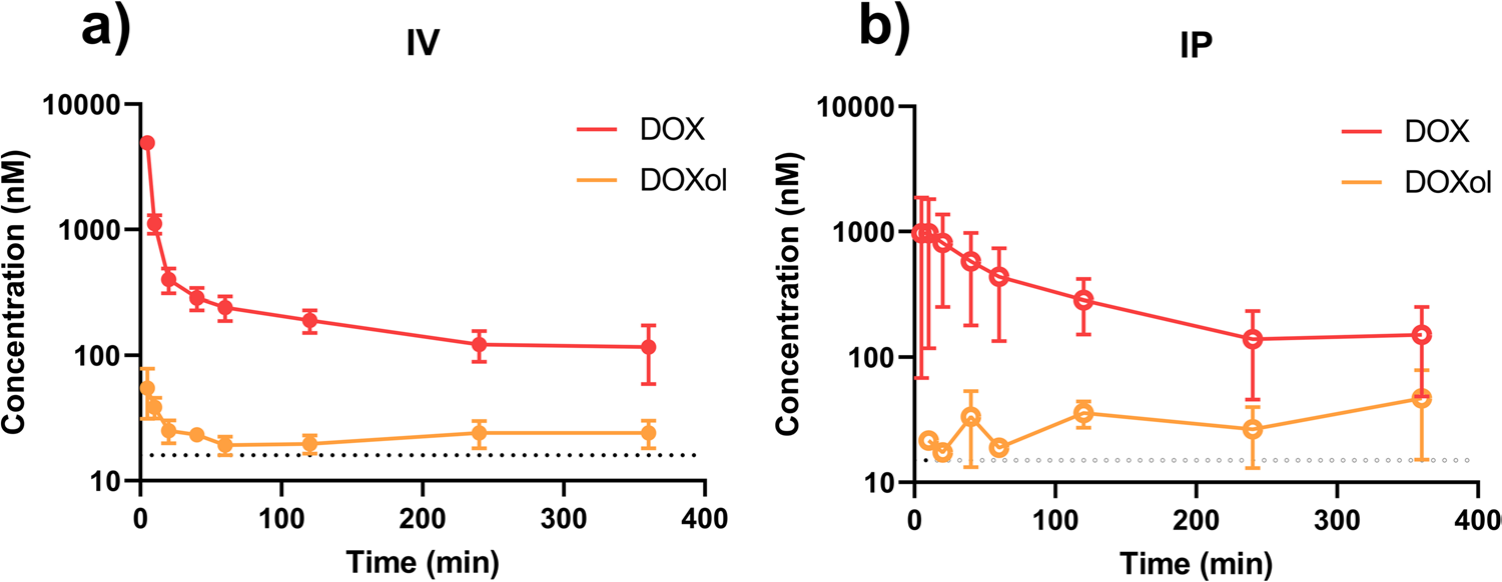
**a**–**b** Mean (± SD) of plasma concentration–time curves for doxorubicin (DOX, red) and doxorubicinol (DOXol, orange). DOX (10 mg/kg) was administered as a single dose (10 mg/kg); **a** intravenous (IV) and **b** intraperitoneal (IP) during 6 h (*n* = 6 rats). Dashed lines denote lowest limit of quantification in the analysis

The plasma concentration–time profile of DOX and its main metabolite DOXol following a single dose IV or IP (10 mg/kg) are displayed in Fig. 4a and b, and the calculated pharmacokinetic parameters of DOX in Table 1. There were no differences in plasma AUC_0–6 h_, t_1/2_, or V_d_ (*p* > 0.05), for the two administration routes.

**Table 1:** Mean (± SD) area under the curve (AUC_0–6 h_), volume of distribution (V_d_), and half-time (t_1/2_) of doxorubicin (DOX, 10 mg/kg) following a single dose injection by IV or IP. The statistical analysis was performed with a student’s *t*-test between IV and IP for each parameter, and none of the differences were significant

	Intravenous (IV)	Intraperitoneal (IP)
AUC_0–6 h_ (nM*min)	76 ± 14	99 ± 53
V_d_ (L/kg)	50 ± 9	44 ± 30
t_1/2_ (min)	200 ± 40	200 ± 80

### Changes in body weight

**Fig. 5:**
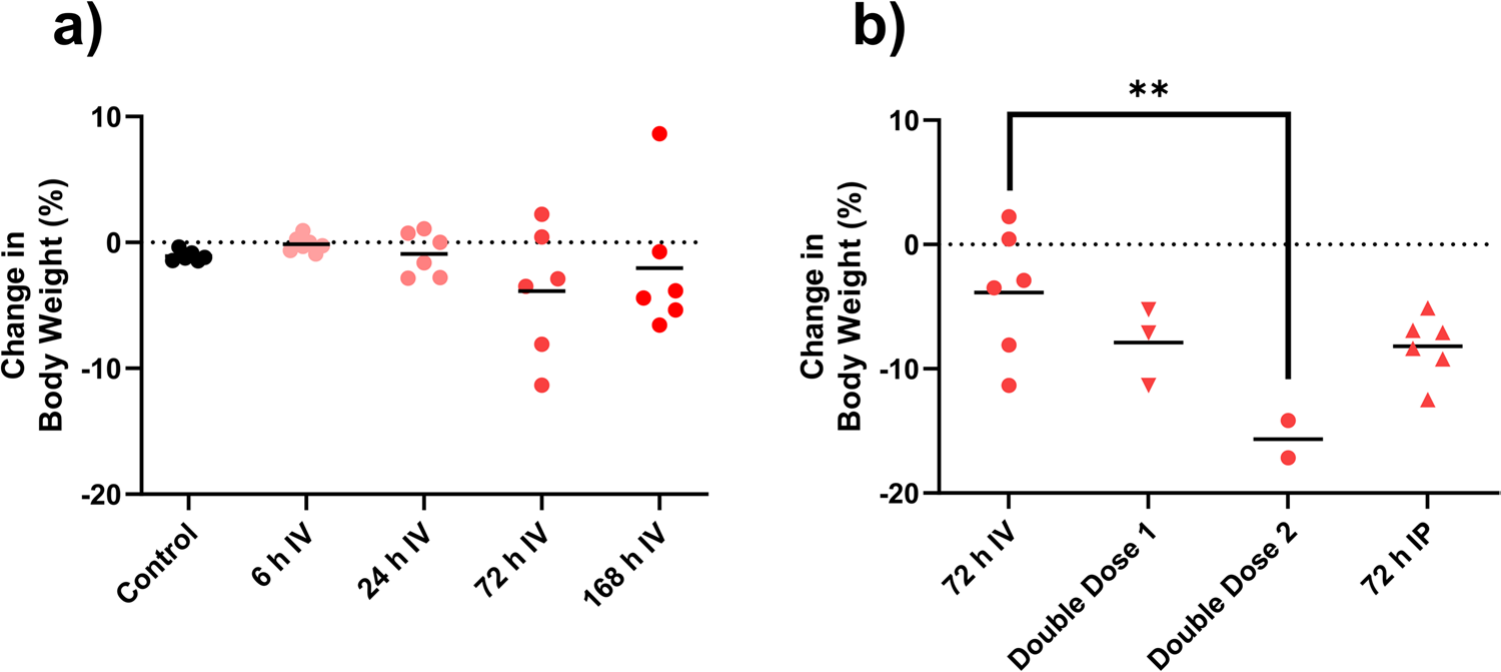
**a**–**b** Changes in body weight after doxorubicin (DOX) doses. Changes over time after a single IV dose of DOX is seen in (**a**), while **b** shows effects of different administration routes (IV and IP) and number of DOX doses (one or two). All DOX was given via IV unless otherwise specified. Each symbol (circles, squares, and triangles) represents the average value from a single animal, and the black line signifies the group average. The statistical data analysis was performed with a Kruskal–Wallis test with Dunn’s multiple comparisons post hoc test, and comparisons with *p* < 0.05 were considered significant, indicated by a star (*p* < 0.05) or two stars (*p* < 0.01)

Body weight was unaffected in all rats that received a single IV or IP dose of DOX (Fig. 5a and b). However, after the two consecutive IV doses 24 h apart, animals evaluated at 96 h after dosing had lost 16% of their body weight compared to baseline (*p* < 0.005) (Fig. 5b).

### Permeability

**Fig. 6:**
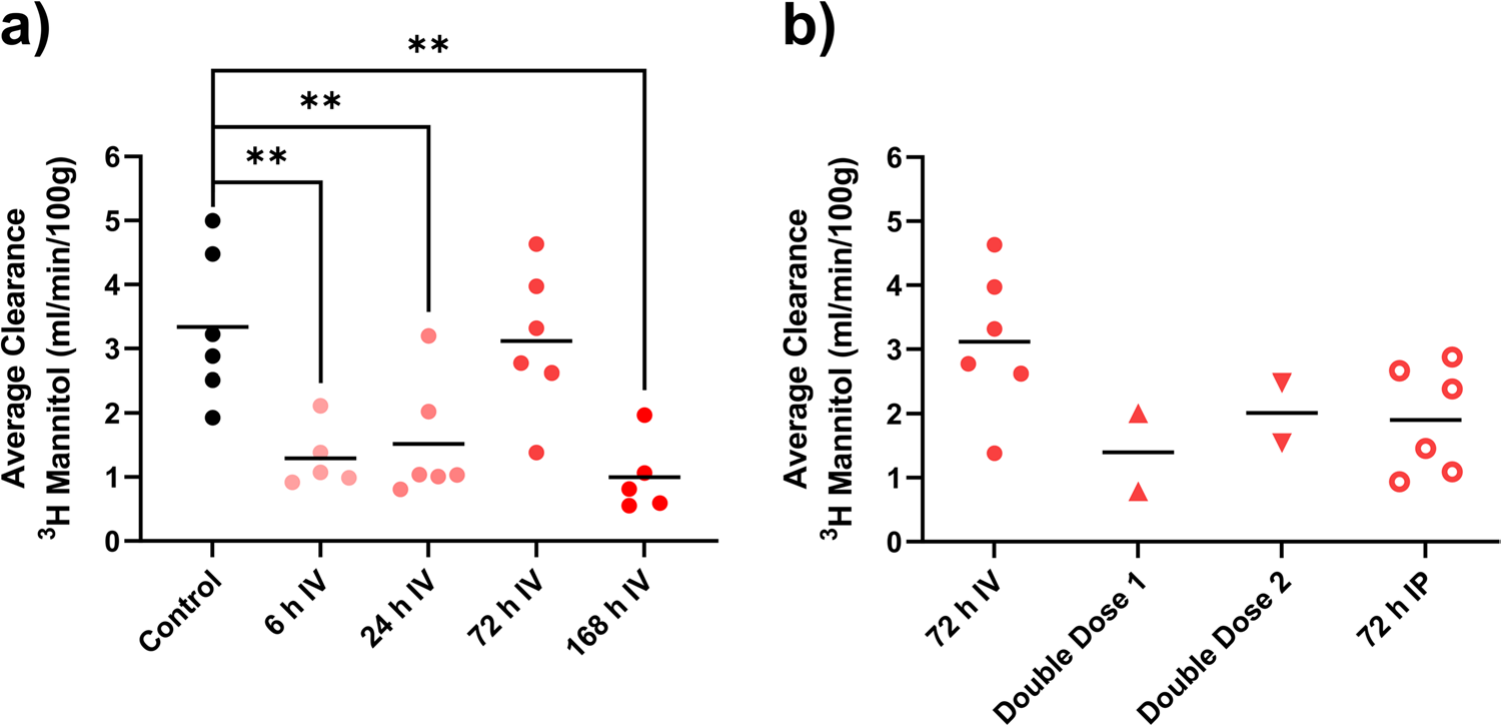
**a**–**b** Jejunal clearance of.^3^H-mannitol was used to reflect mainly paracellular permeability from blood to the single-pass-perfused jejunal segment at different time points after one or two doses of doxorubicin (DOX, 10 mg/kg). Changes over time after a single IV dose of DOX is seen in (**a**), while **b** shows effects of different administration routes (IV and IP) and number of DOX doses (one or two). All DOX was given via IV unless otherwise specified. Each symbol (circles, squares, and triangles) represents the average value from a single animal, and the black line signifies the group average. The statistical data analysis was performed with an ANOVA analysis with Šidák’s multiple comparisons post hoc test, and comparisons with *p* < 0.05 were considered significant, indicated by a star (*p* < 0.05) or two stars (*p* < 0.01)

The intestinal clearance of ^3^H-mannitol from blood-to-lumen (i.e., permeability) was reduced 60% at both 6 and 24 h after DOX IV dosing, compared to the controls (*p* < 0.005). The intestinal clearance then recovered to baseline levels at 72 h, only to be reduced by 70% at 168 h post DOX dose (Fig. 6a). No significant differences in permeability were determined between IV and IP dosing at 72 h, nor between the single or double IV doses (Fig. 6b).

### mRNA expression of marker proteins

**Fig. 7:**
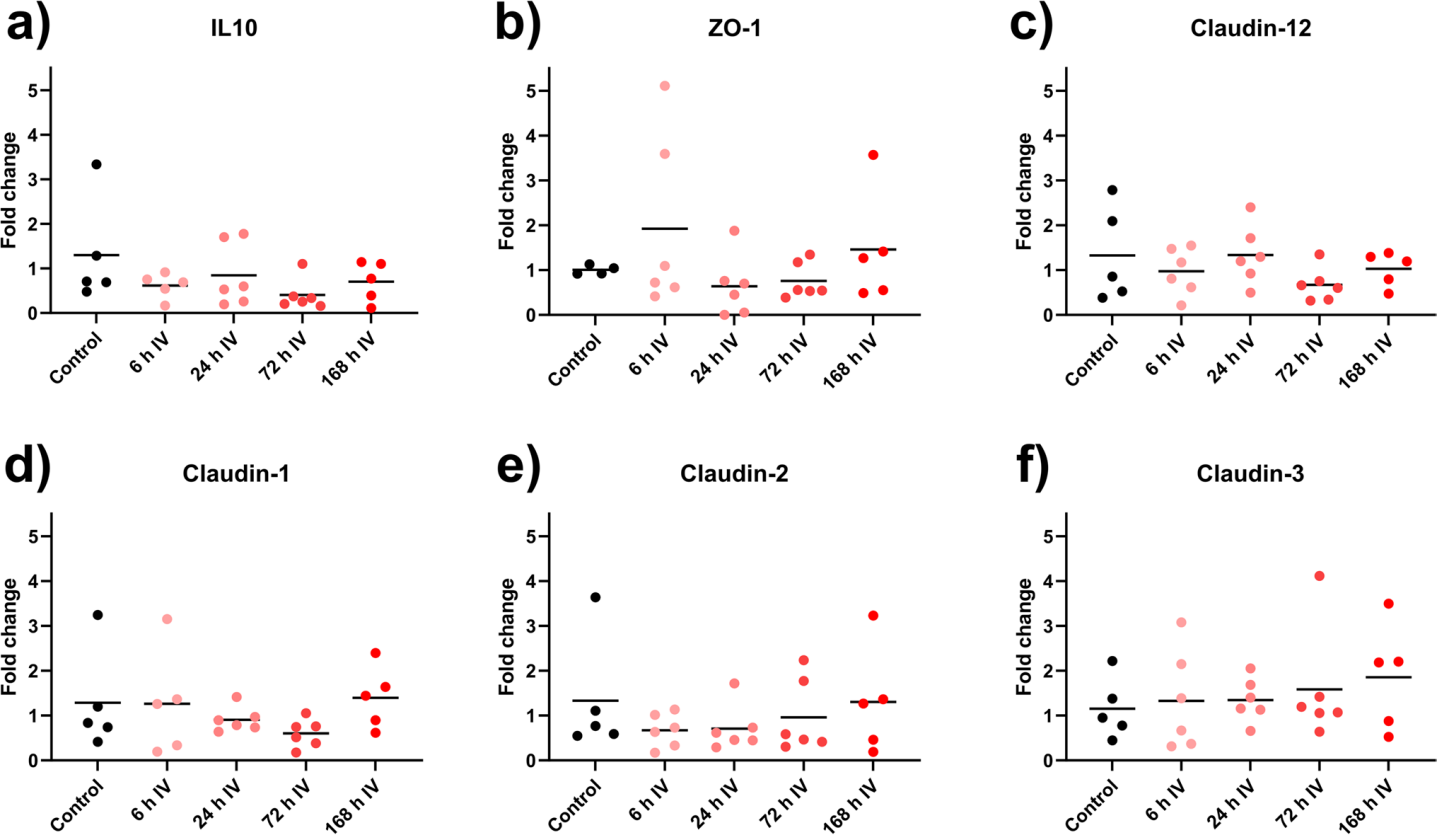
**a**–**f** Change in expression of the six genes IL10 (**a**), ZO-1 (**b**), claudin-12 (**c**), claudin-1 (**d**), claudin 2 (**e**), and claudin-3 (**f**) in jejunjal tissue at four time points following doxorubicin dosing (DOX, 10 mg/kg IV), as determined by RT-qPCR. Each circles represents the average value from a single animal, and the black line signifies the group average. Data are presented as fold change. The statistical data analysis was performed with a Kruskal–Wallis test with Dunn’s multiple comparisons post hoc test, and none of the differences were significant

The mRNA-levels of one anti-inflammatory marker (IL10) and five tight-junction markers (zonula occludens 1 (ZO-1) and claudin 1, 2, 3, and 12) were investigated by RT-qPCR (Fig. 7a–f). Doxorubicin had no clear effect on the expression on any of these markers.

## Discussion

In this in vivo study, we investigated how small intestinal CIM developed over time following a single DOX IV dose. CIM was assayed for functional and morphological changes in the rat jejunum. We also investigated if the administration route (IV vs. IP) of DOX affected any of the measured parameters. We found that peak villus atrophy occurred at 72 h, preceded by a distinct decrease of proliferation and an increase in apoptosis at 24 h after DOX dosing. There was no difference between the IV and IP dosing on villus height reduction and total plasma exposure (AUC_0–6 h_) of DOX.

Chemotherapy-induced villus atrophy is a key morphological effect that can be used for determining the degree of off-target intestinal toxicity (Kaczmarek et al. 2012; Boeing et al. 2021). As such, it is highly relevant to identify an optimal time point for evaluation of protective interventions. In this study, the small intestinal villus height was reduced by 30 to 40% at 72 h after all types of DOX dosing (IV vs. IP and single vs. double dosing). This corroborates previous rodent data, where a 30 to 50% reduction is observed 72 h after 5-fluorouracil (400 mg/kg) or DOX (10 mg/kg) dosing (Kaczmarek et al. 2012; Kissow et al. 2012). Altogether, these results indicated that the most extensive intestinal injury — as determined by morphological change — occurred after 72 h in rodents.

However, the imbalance in apoptosis and proliferation that precedes villus atrophy naturally occurs at earlier time points than the villus atrophy described above (Gehart and Clevers 2019). We showed that Ki67, a proliferation marker, rapidly declined at 24 h after DOX IV dosing. Ki67 levels returned to baseline at 72 h, which agreed with a previous irinotecan (IP, 200 mg/kg) rat study (Al‐Dasooqi et al. 2011). However, later time points also showed a 50% increase of Ki67-stained cells over baseline at 96 h, without fully returning to baseline at 144 h. Combined, this suggests that supportive interventions relying on increased proliferation should ideally be most active 24–48 h after chemotherapy.

In addition to increased proliferation, villus atrophy can be reduced by inhibiting epithelial apoptosis. Our study showed that the average number of apoptotic cells per crypt increased at 24 h post DOX dose, with a non-significant increase of similar magnitude at 6 h; the latter increase possibly reflects a strong inter-individual variability in the onset of apoptosis. While Dekaney et al. (2009) report that DOX mainly causes apoptosis in cells taking positions 3–6 in the crypts, thus belonging to a subset of stem cells that are more prone to cytostatic damage (Dekaney et al. 2009), the method used in this study did not allow for such detailed analysis. Our data corroborate another DOX rat study that reports the highest degree of apoptosis to be at 6 and 24 h after dosing (Kaczmarek et al. 2012). Patient duodenal biopsy data also report a tenfold increase in apoptosis 1 day after standard chemotherapy (Keefe et al. 2000). Together, these data show that apoptosis occurs rapidly after chemotherapy dosing and clearly precedes any visual morphological damages. Any anti-apoptotic supportive treatment should thus focus on the first 24 h after dosing.

Data from human cancer patients (Keefe et al. 2000) and our animal in vivo study indicate that an increase in apoptosis and a decrease in proliferation occur before villus atrophy, while apoptosis and proliferation are back to baseline when villus atrophy is most pronounced. The return to villus height baseline after 168 h is thus most likely related to an increased proliferation occurring after the peak in villus height reduction (Al‐Dasooqi et al. 2011). Another possible explanation is that cell death and shedding of villus tip enterocytes is reduced in the recovery phase. This mechanism has been suggested by others, at least for goblet cells (Verburg et al. 2000). A deeper understanding of the relevant mechanisms and when they occur will be beneficial for the development of supportive treatments that rely on reduced apoptosis and/or increased proliferation.

While previous CIM studies thoroughly investigate mechanisms involved in its development (Sonis 1998; Logan et al. 2009), there is seldom a direct link to functional intestinal processes. One hallmark function of a healthy mucosa is its ability to absorb fluid and nutrients while restricting passage of harmful xenobiotics, viruses, and bacteria (Schoultz and Keita 2020). Intestinal barrier function is often evaluated using low-permeability markers of various sizes, such as mannitol (182 Da) and fluorescein isothiocyanate-dextrans (FITC-dextrans) (> 4 kDa) (Bjarnason et al. 2004). We showed that the proximal small intestinal permeability to the paracellular marker mannitol was reduced at 6, 24, and 168 h after DOX IV dosing. Likewise, there was a downward trend 72 h after IP dosing, concurring with previous IP DOX data from our group (Cano-Cebrián et al. 2022). This is likely related to an immediate sealing of tight-junction complexes, a common mechanism of the intestinal mucosa (Chelakkot et al. 2018) to uphold the barrier function and avoid an uncontrolled “leaky gut” (Camilleri 2019). For instance, tight-junction proteins redistribute to maintain barrier integrity after TNF-induced apoptosis (Marchiando et al. 2011).

Using the Ussing chamber model, Cray et al. have looked at transmembrane flux of FITC-labeled dextrans after DOX exposure to T84 cell monolayers (human colorectal carcinoma) and excised murine jejunal segments. DOX increased the jejunal flux of the 4 kDa dextran in the mouse segments and increased the flux of the 4, 10, and 20 kDa dextrans in the T84 cell monolayer (Cray et al. 2020). This is likely explained by the inability of in vitro systems to utilize the neuroendocrine in vivo feedback response necessary for tight-junction sealing (Hollander and Kaunitz 2020). In vitro systems are thus more sensitive to various chemical challenges than in vivo models, as exemplified by the surfactant sodium dodecyl sulfate which has been demonstrated to be more potent in vitro than in vivo (Dahlgren et al. 2019).

The different results from in vitro and in vivo models may also be related to the choice of permeability probe. Smaller markers (e.g., mannitol) are assumed to be transported across the whole villus axis in charge- and size-selective paracellular pores, while larger ones (FITC-dextrans) utilize the charge- and size-nonselective leak pathway in the basal epithelial layer (Fihn et al. 2000; Buckley and Turner 2018). As such, DOX may increase the permeability only of large probes via the leak pathway. Furthermore, the mice in the Cray et al. study were treated with 20 mg/kg DOX, whereas the T-84 monolayers were exposed to 40 µg/mL, a concentration over 100-fold higher than the average 6 h plasma concentration determined in our study, and 10 times higher than reported T84 IC_50_ values (Jiménez-López et al. 2020). Thus, there may be strong dose-dependent differences in the in vitro and in vivo models with regard to their ability to uphold a functioning mucosal barrier. This highlights the strength of in vivo models for evaluating mucosal functions that rely on the complete neuroendocrine physiological feedback system for full performance. This is further supported by the absence of any effects from DOX on the intestinal permeability of a small marker probe in human cancer patients (Parrilli et al. 1989), in line with the results observed in our rat in vivo study. Still, the difference between in vitro and in vivo methods and the potential role of the protective sealing-off principles in relationship to CIM need further study.

In our study, we could not detect any changes in the mRNA-expression of the tight-junction markers ZO-1 and claudin 1, 2, 3, and 12 following DOX dosing. ZO-1 and other tight-junction proteins contribute to tight-junction barrier function, but neither is essential for basal intestinal epithelial function in vivo (Kuo et al. 2022). The absence of changes in expression may be because RNA transcription of tight-junction proteins has a limited effect on epithelial paracellular permeability (Raleigh et al. 2011; Wardill et al. 2014). It is also possible that CIM-induced changes to the intestinal mucosa are disguised by *submucosa*, *muscularis externa*, and *serosa* tissues that were present in our samples. If possible, RNA experiments should use the isolated mucosa or crypts from the tissue. It also needs to be noted that these data merely reflect transcriptional changes in mRNA-expression of TJP and further analyses are thus needed to assess if protein expression or location of junctional complexes is altered. Our future research regarding cancer drug challenges to the intestinal epithelium will continue to use complementary in vivo and in vitro approaches, such as intestinal organoids, to examine the unresolved issues of tight junction physiology (Kuo et al. 2022; Rodrigues et al. 2022).

Preclinical CIM animal models typically use IP injections (Sun et al. 1998; Dekaney et al. 2009; Kaczmarek et al. 2012; Kissow et al. 2012; Sukhotnik et al. 2014), whereas the main clinical route is IV. Thus, IP injections may possibly cause more extensive intestinal injury due to local drug exposure in the peritoneum. Therefore, we examined if the administration route per se affected jejunal CIM and the plasma pharmacokinetics of DOX. We showed that there was an equal response in villus atrophy, apoptosis, proliferation, and body weight loss, as well as in plasma exposure of DOX for both the IV and IP dosing of the same DOX dose. Overall, our data suggest that local drug exposure in the peritoneum following IP dosing did not increase intestinal toxicity more than what was seen with IV. Rather, systemic exposure seems to be the critical factor for CIM, at least for DOX. Together, this strengthens the relevance of IP dosing in rodent CIM studies. It offers a robust and rapid approach for drug dosing that gives comparable systemic exposure while avoiding any direct, local GI toxicity.

This in vivo study in rats clearly demonstrated time-dependent toxic effects of a single dose of DOX. Villus height was reduced 3 days after DOX dosing, and the intestinal mucosa recovered by day 7. The villus height reduction was preceded by increased apoptosis and decreased proliferation. There was no difference between IV and IP dosing in their effects on various parameters or the plasma pharmacokinetics of DOX. In contrast with in vitro data from the literature, our in vivo model clearly showed that the presence of a neuroendocrine feedback system is crucial for understanding of intestinal barrier function and its dynamic response to tissue challenges such as chemotherapeutics. Furthermore, the choice and combination of permeability markers is important in elucidating precise mechanisms by which intestinal permeability changes. The long-term objective is to use this in vivo model of chemotherapy-induced intestinal mucositis as an experimental tool in the translational development of novel supportive therapies. Based on the experiences in this study, we conclude that the dose of doxorubicin should be at least 10 mg/kg and be given by the IP route as a single dose.

## Supplementary Information

Below is the link to the electronic supplementary material.

Supplementary information A. Detailed description of Ki67 antibody staining. (DOCX 14 KB)

Supplementary information B. Narrative description of image analysis after Ki67 antibody staining. (DOCX 14 KB)

Supplementary information C. ImageJ macro used to quantify the amount of DAB after Ki67 antibody staining. (DOCX 14 KB)

Supplementary information D. R commands used for calculation of NCA. (DOCX 14 KB)

Table S1. Primer sequences for genes used during RT-qPCR. (XLSX 9 KB)

Table S2. Raw data used for NCA analysis. (XLSX 11 KB)

## Data Availability

The data presented in this study are available on request from the corresponding author.

## References

[CR1] Al-Dasooqi N, Bowen JM, Gibson RJ, Logan RM, Stringer AM, Keefe DM (2011). Irinotecan-induced alterations in intestinal cell kinetics and extracellular matrix component expression in the dark agouti rat. Int J Exp Pathol.

[CR2] Bjarnason I, Takeuchi K, Bjarnason A, Adler S, Teahon K (2004). The GUT of gut. Scand J Gastroenterol.

[CR3] Boeing T, Gois MB, de Souza P, Somensi LB, da Silva LM (2021). Irinotecan-induced intestinal mucositis in mice: a histopathological study. Cancer Chemother Pharmacol.

[CR4] Boschi R, Rostagno E (2012). Extravasation of antineoplastic agents: prevention and treatments. Pediatric Reports.

[CR5] Bowen J, Al-Dasooqi N, Bossi P, Wardill H, Van Sebille Y, Al-Azri A, Bateman E, Correa M, Raber-Durlacher J, Kandwal A (2019). The pathogenesis of mucositis: updated perspectives and emerging targets. Support Care Cancer.

[CR6] Bray F, Laversanne M, Weiderpass E, Soerjomataram I (2021). The ever-increasing importance of cancer as a leading cause of premature death worldwide. Cancer.

[CR7] Buckley A, Turner JR (2018). Cell biology of tight junction barrier regulation and mucosal disease. Cold Spring Harb Perspect Biol.

[CR8] Camilleri M (2019). Leaky gut: mechanisms, measurement and clinical implications in humans. Gut.

[CR9] Cano-Cebrián M-J, Dahlgren D, Kullenberg F, Peters K, Olander T, Sjöblom M, Lennernäs H (2022). Chemotherapeutics combined with luminal irritants: effects on small-intestinal mannitol permeability and villus length in rats. Int J Mol Sci.

[CR10] Chelakkot C, Ghim J, Ryu SH (2018). Mechanisms regulating intestinal barrier integrity and its pathological implications. Exp Mol Med.

[CR11] Cray P, Sheahan BJ, Cortes JE, Dekaney CM (2020). Doxorubicin increases permeability of murine small intestinal epithelium and cultured T84 monolayers. Sci Rep.

[CR12] Dahlgren D, Sjöblom M, Lennernäs H (2019) Intestinal absorption-modifying excipients: a current update on preclinical in vivo evaluations. Eur J Pharm Biopharm pp 142411–420. 10.1016/j.ejpb.2019.07.013 10.1016/j.ejpb.2019.07.013 31306749

[CR13] Dahlgren D, Olander T, Sjöblom M, Hedeland M, Lennernäs H (2021). Effect of paracellular permeation enhancers on intestinal permeability of two peptide drugs, enalaprilat and hexarelin, in rats. Acta Pharmaceutica Sinica B.

[CR14] Dahlgren D, Sjöblom M, Hellström PM, Lennernäs H (2021). Chemotherapeutics-induced intestinal mucositis: pathophysiology and potential treatment strategies. Front Pharmacol.

[CR15] Dahlgren D, Rosenqvist E, Hellström PM, Nygren P, Kullenberg F, Peters K, Sjöblom M, Lennernäs H (2022) Evaluation and validation of chemotherapy‐specific diarrhoea and histopathology in rats. Basic Clin Pharmacol Toxicol. 10.1111/bcpt.13790 10.1111/bcpt.13790 PMC9828157 36124882

[CR16] Dekaney CM, Gulati AS, Garrison AP, Helmrath MA, Henning SJ (2009). Regeneration of intestinal stem/progenitor cells following doxorubicin treatment of mice. Am J Physiol Gastrointest Liver Physiol.

[CR17] Eduardo FP, Bezinelli LM, Gobbi M, Rosin FC, Carvalho DL, Ferreira MH, da Silva CC, Hamerschlak N, Corrêa L (2019). Retrospective study of the digestive tract mucositis derived from myeloablative and non-myeloablative/reduced-intensity conditionings with busulfan in hematopoietic cell transplantation patient. Support Care Cancer.

[CR18] Fihn BM, Sjöqvist A, Jodal M (2000). Permeability of the rat small intestinal epithelium along the villus-crypt axis: effects of glucose transport. Gastroenterology.

[CR19] Gavrieli Y, Sherman Y, Ben-Sasson SA (1992). Identification of programmed cell death in situ via specific labeling of nuclear DNA fragmentation. J Cell Biol.

[CR20] Gehart H, Clevers H (2019). Tales from the crypt: new insights into intestinal stem cells. Nat Rev Gastroenterol Hepatol.

[CR21] Gewirtz D (1999). A critical evaluation of the mechanisms of action proposed for the antitumor effects of the anthracycline antibiotics adriamycin and daunorubicin. Biochem Pharmacol.

[CR22] Gibson RJ, Bowen JM, Inglis MR, Cummins AG, Keefe DM (2003). Irinotecan causes severe small intestinal damage, as well as colonic damage, in the rat with implanted breast cancer. J Gastroenterol Hepatol.

[CR23] Hollander D, Kaunitz JD (2020). The “leaky gut”: tight junctions but loose associations?. Dig Dis Sci.

[CR24] Jiménez-López J, García-Hevia L, Melguizo C, Prados J, Bañobre-López M, Gallo J (2020). Evaluation of novel doxorubicin-loaded magnetic wax nanocomposite vehicles as cancer combinatorial therapy agents. Pharmaceutics.

[CR25] Jones RL, Wagner AJ, Kawai A, Tamura K, Shahir A, Van Tine BA, Martín-Broto J, Peterson PM, Wright J, Tap WD (2021). Prospective evaluation of doxorubicin cardiotoxicity in patients with advanced soft-tissue sarcoma treated in the ANNOUNCE phase III randomized trial. Clin Cancer Res.

[CR26] Kaczmarek A, Brinkman BM, Heyndrickx L, Vandenabeele P, Krysko DV (2012). Severity of doxorubicin-induced small intestinal mucositis is regulated by the TLR-2 and TLR-9 pathways. J Pathol.

[CR27] Keefe D, Brealey J, Goland G, Cummins A (2000). Chemotherapy for cancer causes apoptosis that precedes hypoplasia in crypts of the small intestine in humans. Gut.

[CR28] Kissow H, Viby N-E, Hartmann B, Holst JJ, Timm M, Thim L, Poulsen SS (2012). Exogenous glucagon-like peptide-2 (GLP-2) prevents chemotherapy-induced mucositis in rat small intestine. Cancer Chemother Pharmacol.

[CR29] Krugliak P, Hollander D, Schlaepfer C, Nguyen H, Ma T (1994). Mechanisms and sites of mannitol permeability of small and large intestine in the rat. Dig Dis Sci.

[CR30] Kullenberg F, Degerstedt O, Calitz C, Pavlović N, Balgoma D, Gråsjö J, Sjögren E, Hedeland M, Heindryckx F, Lennernäs H (2021). In vitro cell toxicity and intracellular uptake of doxorubicin exposed as a solution or liposomes: implications for treatment of hepatocellular carcinoma. Cells.

[CR31] Kuo WT, Odenwald MA, Turner JR, Zuo L (2022). Tight junction proteins occludin and ZO-1 as regulators of epithelial proliferation and survival. Ann N Y Acad Sci.

[CR32] Logan RM, Stringer AM, Bowen JM, Gibson RJ, Sonis ST, Keefe DM (2009). Is the pathobiology of chemotherapy-induced alimentary tract mucositis influenced by the type of mucotoxic drug administered?. Cancer Chemother Pharmacol.

[CR33] Marchiando AM, Shen L, Graham WV, Edelblum KL, Duckworth CA, Guan Y, Montrose MH, Turner JR, Watson AJM (2011) The epithelial barrier is maintained by in vivo tight junction expansion during pathologic intestinal epithelial shedding. Gastroenterology 140(4):1208–1218. 10.1053/j.gastro.2011.01.004 10.1053/j.gastro.2011.01.004 PMC3066304 21237166

[CR34] Marinello J, Delcuratolo M, Capranico G (2018). Anthracyclines as topoisomerase II poisons: from early studies to new perspectives. Int J Mol Sci.

[CR35] McCullough RW (2017). US oncology-wide incidence, duration, costs and deaths from chemoradiation mucositis and antimucositis therapy benefits. Futur Oncol.

[CR36] Mirzayans R, Murray D (2020). Do TUNEL and other apoptosis assays detect cell death in preclinical studies?. Int J Mol Sci.

[CR37] Nair AB, Jacob S (2016). A simple practice guide for dose conversion between animals and human. J Basic Clin Pharm.

[CR38] Nylander O, Kvietys P, Granger DN (1989). Effects of hydrochloric acid on duodenal and jejunal mucosal permeability in the rat. Am J Physiol Gastrointest Liver Physiol.

[CR39] Nylander O, Sababi M, Bark J (1991). Characterization of 51Cr-EDTA as a marker of duodenal mucosal permeability. Acta Physiol Scand.

[CR40] Parrilli G, Iaffaioli RV, Martorano M, Cuomo R, Tafuto S, Zampino MG, Budillon G, Raffaele Bianco A (1989). Effects of anthracycline therapy on intestinal absorption in patients with advanced breast cancer. Can Res.

[CR41] Pico JL, Avila-Garavito A, Naccache P (1998). Mucositis: its occurrence, consequences, and treatment in the oncology setting. Oncologist.

[CR42] Raleigh DR, Boe DM, Yu D, Weber CR, Marchiando AM, Bradford EM, Wang Y, Wu L, Schneeberger EE, Shen L, Turner JR (2011) Occludin S408 phosphorylation regulates tight junction protein interactions and barrier function. J Cell Biol 193(3):565–582. 10.1083/jcb.201010065 10.1083/jcb.201010065 PMC3087007 21536752

[CR43] Reilly JJ, Neifeld JP, Rosenberg SA (1977). Clinical course and management of accidental adriamycin extravasation. Cancer.

[CR44] Rodrigues D, Coyle L, Füzi B, Ferreira S, Jo H, Herpers B, Chung S-W, Fisher C, Kleinjans JC, Jennen D (2022). Unravelling mechanisms of doxorubicin-induced toxicity in 3D human intestinal organoids. Int J Mol Sci.

[CR45] Rodrigues-Oliveira L, Kowalski LP, Santos M, Marta GN, Bensadoun R-J, Martins MD, Lopes MA, de Castro JG, William WN, Chaves ALF (2021). Direct costs associated with the management of mucositis: a systematic review. Oral Oncol.

[CR46] Schoultz I, Keita ÅV (2020). The intestinal barrier and current techniques for the assessment of gut permeability. Cells.

[CR47] Sonis S (1998). Mucositis as a biological process: a new hypothesis for the development of chemotherapy-induced stomatotoxicity. Oral Oncol.

[CR48] Sonis ST (2009). Mucositis: the impact, biology and therapeutic opportunities of oral mucositis. Oral Oncol.

[CR49] Sonis S, Elting L, Keefe D, Nguyen H, Grunberg S, Randolph-Jackson P, Brennan M (2015). Unanticipated frequency and consequences of regimen-related diarrhea in patients being treated with radiation or chemoradiation regimens for cancers of the head and neck or lung. Support Care Cancer.

[CR50] Sougiannis AT, VanderVeen BN, Davis JM, Fan D, Murphy EA (2021). Understanding chemotherapy-induced intestinal mucositis and strategies to improve gut resilience. Am J Physiol Gastroint Liver Physiol.

[CR51] Sukhotnik I, Pollak Y, Coran AG, Pilatov J, Bejar J, Mogilner JG, Berkowitz D (2014). Glutamine attenuates the inhibitory effect of methotrexate on TLR signaling during intestinal chemotherapy-induced mucositis in a rat. Nutr Metab.

[CR52] Sun Z, Wang X, Wallen R, Deng X, Du X, Hallberg E, Andersson R (1998). The influence of apoptosis on intestinal barrier integrity in rats. Scand J Gastroenterol.

[CR53] Sung H, Ferlay J, Siegel RL, Laversanne M, Soerjomataram I, Jemal A, Bray F (2021). Global cancer statistics 2020: GLOBOCAN estimates of incidence and mortality worldwide for 36 cancers in 185 countries. CA: Cancer J Clin.

[CR54] Tonneau M, Elkrief A, Pasquier D, Del Socorro TP, Chamaillard M, Bahig H, Routy B (2021). The role of the gut microbiome on radiation therapy efficacy and gastrointestinal complications: a systematic review. Radiother Oncol.

[CR55] van der Zanden SY, Qiao X, Neefjes J (2020). New insights into the activities and toxicities of the old anticancer drug doxorubicin. FEBS J.

[CR56] Verburg M, Renes IB, Meijer HP, Taminiau JA, Büller HA, Einerhand AW, Dekker J (2000). Selective sparing of goblet cells and paneth cells in the intestine of methotrexate-treated rats. Am J Physiol Gastrointest Liver Physiol.

[CR57] Wardill HR, Gibson RJ, Logan RM, Bowen JM (2014) TLR4/PKC-mediated tight junction modulation: a clinical marker of chemotherapy-induced gut toxicity?. Int J Cancer 135(11):2483–2492. 10.1002/ijc.28656 10.1002/ijc.28656 24310924

