# Supplementary material for: The progression of doxorubicin-induced intestinal mucositis in rats
Source: Naunyn Schmiedebergs Arch Pharmacol. 2022 Oct 22;396(2):247–60. doi: 10.1007/s00210-022-02311-6 (PMC9832110; doi:10.1007/s00210-022-02311-6)
Supplement: Supplementary file 1 — Supplementary information A. Detailed description of Ki67 antibody staining. (DOCX 14 KB) [file 210_2022_2311_MOESM1_ESM.docx]

Detection of proliferation was performed using Ki67 antibody (Abcam, ab16667, Cambridge, UK) with a horseradish peroxidase - DAB detection IHC kit from Abcam (ab64261, Abcam, Cambridge, UK). Slides from each treatment category were taken, as well as a negative control to investigate unspecific binding. Prior to staining, deparaffinization was carried out, followed by washing with PBS buffer with Tween for 2 min, before adding drops of Hydrogen Peroxide Block for 10 min. A DIVA-de-cloaking chamber was used to retrieve cross-linked antigens. Afterwards, sections were washed 3 times in the PBS buffer, dried and samples were circled using a Histo-Pen (Liquid-Blocker). Protein Block was then applied on the slides, and incubated for 15 min at room temperature to prevent nonspecific background staining. Primary Transferrin Ki67 antibody (Abcam ab16667, Cambridge, UK) was added in a 1:1000 dilution of PBS-Tween, followed by a one hour incubation at 37°C. Then, the slides were rinsed 4 times in the PBS buffer prior to the application of biotinylated goat anti-rabbit antibodies and incubated at room temperature for 10 min and washed again 4 times in the PBS buffer. Streptavidin Peroxidase was applied on the sections before incubating them again for 10 min at room temperature and rinsing them 4 times in PBS. 20 µl DAB Chromogen to 1ml of DAB Substrate were mixed by swirling and were then added and applied to the jejunum tissues, prior to being incubated for exactly 2 min and rinsed again 4 times in the PBS buffer. Thereafter, the slides were immersed in hematoxylin and incubated for 3 min followed by 10 minutes of washing in lukewarm running tap water. Afterwards, the slides were dehydrated and mounted. Images were acquired using a Zeiss Axio Vert microscope equipped with a Zeiss Axiocam 208 color camera and Zeiss A-Plan 10x/0,25 Ph1 objective. The Zeiss Zen Blue 4.3 software was used to save and transfer the pictures.
