# Supplementary material for: The progression of doxorubicin-induced intestinal mucositis in rats
Source: Naunyn Schmiedebergs Arch Pharmacol. 2022 Oct 22;396(2):247–60. doi: 10.1007/s00210-022-02311-6 (PMC9832110; doi:10.1007/s00210-022-02311-6)
Supplement: Supplementary file 2 — Supplementary information B. Narrative description of image analysis after Ki67 antibody staining. (DOCX 14 KB) [file 210_2022_2311_MOESM2_ESM.docx]

The images were processed using in an ImageJ macro to automatically quantify the amount of DAB staining. The first step was to quantify the amount of sample in the image by converting it to grayscale and using a threshold of (0, 210) to determine how much of the image contained sample. To determine how much was stained with DAB, the inbuilt deconvolution tool was used to separate DAB and hematoxylin in the original image, and the DAB image was quantified using a second threshold of (0,100).To calculate a percentage of the stained sample, a ratio between the two was calculated. See Figure S1 for a graphical demonstration of this process.

To correct for background staining, a section of each image containing only the upper part of the villi was selected, as no proliferation was expected in this region. These sections were then quantified as described above. The final value for each image was determined as the stained sample from the full image minus the stained sample from the control image.

In this analysis there were two cases (one at 168 hours exposure in Figure 5a and one at 48+72 hours exposure in Figure 5b) where the background images contained more staining per sample than the full images, leading to a calculated negative area that was stained after the correction. In these cases, the value was manually set to zero in both Figure 5 and the statistical analysis.
