# Supplementary material for: The progression of doxorubicin-induced intestinal mucositis in rats
Source: Naunyn Schmiedebergs Arch Pharmacol. 2022 Oct 22;396(2):247–60. doi: 10.1007/s00210-022-02311-6 (PMC9832110; doi:10.1007/s00210-022-02311-6)
Supplement: Supplementary file 3 — Supplementary information C. ImageJ macro used to quantify the amount of DAB after Ki67 antibody staining. (DOCX 14 KB) [file 210_2022_2311_MOESM3_ESM.docx]

#@ File (label = "Input directory", style = "directory") input

#@ File (label = "Output directory", style = "directory") output

#@ String (label = "File suffix", value = ".tif") suffix

processFolder(input);

// function to scan folders/subfolders/files to find files with correct suffix

function processFolder(input) {

list = getFileList(input);

list = Array.sort(list);

for (i = 0; i < list.length; i++) {

if(File.isDirectory(input + File.separator + list[i]))

processFolder(input + File.separator + list[i]);

if(endsWith(list[i], suffix))

processFile(input, output, list[i]);

}

}

function processFile(input, output, file) {

open(file);

run("Colour Deconvolution", "vectors=[H DAB]");

windowname = file+"-(Colour_2)";

selectWindow(windowname); //select the image with only DAB staining

setAutoThreshold("Default");

//run("Threshold...") //to mark what is sample on image;

setThreshold(0, 210);

setOption("BlackBackground", false);

run("Convert to Mask");

run("Measure");

saveAs("tiff", output+file);

close("*");

open(file);

run("Set Scale...", "distance=2.7 known=1 unit=µm");

run("Colour Deconvolution", "vectors=[H DAB]");

windowname = file+"-(Colour_2)";

selectWindow(windowname);

setAutoThreshold("Default");

//run("Threshold...");

setThreshold(0, 100);

setOption("BlackBackground", false);

run("Convert to Mask");

run("Measure"); //get the percent sample on image

saveAs("png", output+file);

close("*");

}
