# Supplementary material for: The progression of doxorubicin-induced intestinal mucositis in rats
Source: Naunyn Schmiedebergs Arch Pharmacol. 2022 Oct 22;396(2):247–60. doi: 10.1007/s00210-022-02311-6 (PMC9832110; doi:10.1007/s00210-022-02311-6)
Supplement: Supplementary file 4 — Supplementary information D. R commands used for calculation of NCA. (DOCX 14 KB) [file 210_2022_2311_MOESM4_ESM.docx]

1. R settings used for calculation of NCA:

library(dplyr)

library(magrittr)

library(knitr)

library(ncappc)

library(readxl)

DOX_IP_IV <- read_excel("C:/Users/freku365/Downloads/DOX_IP_IV.xlsx",

col_types = c("text", "numeric", "numeric", "numeric"))

out <- ncappc(

obsFile=DOX_IP_IV,

concUnit = "nM",

doseUnit = "nmol/kg",

timeUnit = "minutes",

adminType = "extravascular",

outFileNm = "DOX_IP_IV",

onlyNCA = T,

extrapolate = T,

printOut = F,

evid = F,

noPlot = T

)
